# TRPP2 dysfunction decreases ATP-evoked calcium, induces cell aggregation and stimulates proliferation in T lymphocytes

**DOI:** 10.1186/s12882-019-1540-6

**Published:** 2019-09-13

**Authors:** Riccardo Magistroni, Alessandra Mangolini, Sonia Guzzo, Francesca Testa, Mario R. Rapanà, Renzo Mignani, Giorgia Russo, Francesco di Virgilio, Gianluca Aguiari

**Affiliations:** 1 Surgical, Medical and Dental Department of Morphological Sciences related to Transplant, Oncology and Regenerative Medicine, University of Modena and Reggio Emilia, Azienda Opedaliero-Universitaria di Modena, Largo del Pozzo, Modena, Italy; 2 Department of Biomedical and Surgical Specialty Sciences, University of Ferrara, via Luigi Borsari 46, 44100 Ferrara, Italy; 3 Unità Operativa di Nefrologia e Dialisi, Azienda USL Ospedale Santa Maria della Scaletta di Imola, via Montericco 4, Imola, Italy; 4 Unità Operativa di Nefrologia e Dialisi, Azienda AUSL Ospedale degli Infermi di Rimini, viale Luigi Settembrini 2, Rimini, Italy; 5 Unità Operativa di Nefrologia e Dialisi, Azienda Ospedaliero Universitaria Arcispedale Sant’Anna di Ferrara, via Aldo Moro 8, Ferrara, Italy; 6 Department of Morphology, Surgery and Experimental Medicine, University of Ferrara, via Luigi Borsari 46, Ferrara, Italy

**Keywords:** ADPKD, TRPP2, Calcium, mTOR, ERK, NFkB, T lymphocytes

## Abstract

**Background:**

Autosomal dominant polycystic kidney disease (ADPKD) is mainly characterised by the development and enlargement of renal cysts that lead to end-stage renal disease (ESRD) in adult patients. Other clinical manifestations of this pathology include hypertension, haematuria, abdominal pain, cardiovascular system alterations and intracranial aneurysms. ADPKD is linked to mutations in either *PKD1* or *PKD2* that codifies polycystin-1 (PC1) and polycystin-2 (PC2 or TRPP2), respectively. PC1 and TRPP2 are membrane proteins that function as receptor-channel elements able to regulate calcium homeostasis. The function of polycystins has been mainly studied in kidney cells; but the role of these proteins in T lymphocytes is not well defined.

**Methods:**

T lymphocytes were produced from ADPKD1 and ADPKD2 patients as well as from non-ADPKD subjects undergoing renal replacement therapy (RRT) and healthy controls. Protein expression and phosphorylation levels were analysed by western blotting, cell proliferation was calculated by direct counting using trypan blue assay and intracellular calcium concentration was measured by Fura-2 method.

**Results:**

*PKD2* mutations lead to the significant reduction of TRPP2 expression in T lymphocytes derived from ADPKD patients. Furthermore, a smaller TRPP2 truncated protein in T lymphocytes of patients carrying the mutation R872X in *PKD2* was also observed, suggesting that TRPP2 mutated proteins may be stably expressed. The silencing or mutation of *PKD2* causes a strong reduction of ATP-evoked calcium in Jurkat cells and ADPKD2 T lymphocytes, respectively. Moreover, T lymphocytes derived from both ADPKD1 and ADPKD2 patients show increased cell proliferation, basal chemotaxis and cell aggregation compared with T lymphocytes from non-ADPKD subjects. Similarly to observations made in kidney cells, mutations in *PKD1* and *PKD2* dysregulate ERK, mTOR, NFkB and MIF pathways in T lymphocytes.

**Conclusions:**

Because the alteration of ERK, mTOR, NFkB and MIF signalling found in T lymphocytes of ADPKD patients may contribute to the development of interstitial inflammation promoting cyst growth and kidney failure (ESRD), the targeting of inflammasome proteins could be an intriguing option to delay the progression of ADPKD.

**Electronic supplementary material:**

The online version of this article (10.1186/s12882-019-1540-6) contains supplementary material, which is available to authorized users.

## Background

Autosomal dominant polycystic kidney disease (ADPKD) is a chronic and progressive pathology characterised by the formation and growth of fluid-filled cysts in the kidney that in adult life lead to end-stage renal disease (ESRD) [1]. The formation and enlargement of kidney cysts is driven by alterations in epithelial cell growth, fluid secretion and extracellular matrix composition [1].

ADPKD is a systemic disorder that includes a variety of extra-renal clinical complications, such as abdominal pain, hepatic and pancreatic cysts, hypertension, valvular heart abnormalities and intracranial aneurysms [2, 3]. Currently, cardiovascular complications and intracranial aneurysms are the main cause of death in ADPKD patients [2]. It is caused by mutations in *PKD1* and *PKD2* genes. However, the focal development of kidney cysts occurs by a process defined “two-hit” where the first event is represented by the germline mutation, whereas the somatic inactivation of the normal *PKD1* or *PKD2* allele constitutes the second hit [4]. *PKD1* and *PKD2* encode for polycystin-1 (PC1) and polycystin-2 (PC2 or TRPP2), respectively. PC1 is a membrane protein that is also expressed in the primary cilium of kidney cells, where it interacts with TRPP2 and is able to regulate calcium entry [1]. TRPP2 is a member of the transient receptor potential channel family and functions as a non-selective calcium channel protein [5]. Polycystins are expressed in different tissues including kidney, vasculature, cardiomyocytes and B lymphocytes [3, 6]. These proteins interact with each other by forming complexes involved in the modulation of different signalling pathways, such as the B-Raf/MEK/ERK cascade, mTOR kinase and EGF receptor, regulating the differentiation, growth and apoptosis of kidney cells [7]. However, the functions of polycystins in other cell types are not well known. The expression of PC1 and TRPP2 in smooth muscle cells supports their involvement in the maintenance of the myoelastic structure of arteries [8]. Moreover, the loss of TRPP2 function could impair the directional cell migration in the lymphatic vasculature, resulting in reduced vessel density [9]. TRPP2 mutation also seems to be associated with idiopathic dilated cardiomyopathy in ADPKD patients, which is likely due to abnormal intracellular calcium cycling [2]. The risk for cardiovascular mortality in ADPKD is increased by chronic inflammation; moreover, inflammatory processes contribute to disease progression, which is dependent on polycystic altered signalling [10]. In this regard, the innate immune system and inflammation could be potential therapeutic targets to slow disease progression. Inflammatory processes are mediated by the activation of Toll-like receptors (TLRs) that play a role in recognising pathogen-associated molecular patterns and promote the activation of leucocytes [11]. Therefore, immune cells could play an important role in the progression of ADPKD, modulating the inflammatory response and immune system. However, the expression and function of polycystins in blood cells is poorly studied, especially the role of these proteins in T lymphocytes.

Here, we have investigated TRPP2 expression, calcium release after ATP and PAF stimulation as well as signalling pathways involved in the inflammation in T lymphocytes generated from ADPKD patients.

## Methods

### Materials

Culture media, saline buffers and plastic material were obtained from EuroClone (Milan, Italy). Anti-P-mTOR, anti-mTOR, anti-P-ERK, anti-ERK, and anti-MIF antibodies were purchased from Cell Signaling Technology (EuroClone). Anti PC1 7E12, anti-β-actin and anti-NFkB antibodies were bought from Santa Cruz Biotechnology (Milan, Italy). Anti-CD3 (OKT3) monoclonal antibody was purchased from Invitrogen (Thermo Fisher Scientific, Milan, Italy). The anti-PC2 N-ter antibody was kindly provided by Prof. Stefan Somlo (Yale University, CT, USA). Enhanced chemiluminescent substrates for Western blotting, HRP-conjugated goat anti-rabbit and anti-mouse antibodies were purchased from Cell Signaling Technology (EuroClone). Lympholyte®-H and phytohaemagglutinin (PHA) were obtained from Cedarlane Laboratories (Tebu-Bio, Milan, Italy) and EuroClone, respectively. Interleukin-2 (IL-2) and Fura 2-AM were purchased from Invitrogen. Platelet-activating factor (PAF), ATP and ionomycin were purchased from Sigma-Aldrich (Milan, Italy). The recombinant plasmid for *PKD2* silencing (TRPP2-siRNA) was constructed in our laboratory by using the pSuper vector (Oligoengine, Seattle, WA) as previously described [12].

### Patients and controls

This study was conducted analysing 110 ADPKD patients (30 ADPKD1, 21 ADPKD2 and 59 ADPKD not genetically determined). As control samples, 21 non-ADPKD subjects undergoing renal replacement therapy (RRT)^1^ and 27 healthy controls were used. Peripheral blood samples, 20–30 mL, were collected in EDTA vacuum tubes from four hospital units of Emilia Romagna Region (Italy). Clinical features of ADPKD patients are indicated in Additional file 1: Table S1. The study was performed in accordance with the guidelines of the Helsinki Declaration.

### Isolation of T lymphocytes and neutrophils

Peripheral blood lymphocytes (PBLs) were isolated by lympholyte-H cell separation density gradient. Next, PBLs were resuspended in RPMI 1640 supplemented with 10% FBS and separated from PBMC by 2 h of adherence in a T25 flask to remove monocytes. Cells were collected and cultured in RPMI medium containing 10% FBS supplemented with 2 μg/mL PHA for 72 h. Finally, T lymphocytes were grown for at least 72 h in RPMI 10% FBS medium supplemented with 50 U/mL of IL-2.

Human polymorphonuclear neutrophils (PMNs) were isolated from the blood of ADPKD and control samples by the removal of red blood cells (RBCs) using dextran-promoted rosette formation and hypotonic lysis of remaining RBCs. PMNs were separated from PBMC stratifying the cells on a Percoll-based density gradient (Sigma-Aldrich, Milan, Italy), as previously described [13]. Isolated cells were washed twice in Krebs-Ringer-phosphate buffer containing 0.1% w/v glucose, pH 7.4 (KRPG) and resuspended at a concentration of 5 × 10^7^ cells/mL. Next, cells were maintained at room temperature until the examination. By this method, neutrophils can be isolated with > 95% purity [13]. The percentage of viable cells was > 99% as determined by the trypan blue exclusion test.

### Cell transfection

Jurkat cells were transiently transfected with a TRPP2-siRNA plasmid, expressing specific *PKD2* silencing sequences [12], by using the TransIT®-Jurkat Transfection Reagent (Mirus Bio, Tema Ricerca, Bologna, Italy). Briefly, cells were resuspended at 5 × 10^5^ cell density in 500 μL of complete medium and stratified on a 100 μL serum-free medium containing 1 μg of plasmid and 3 μL of transfection reagent. After 5 h of incubation, cells were pelleted by centrifugation at 800 *g* for 5 min and resuspended in fresh complete medium. As control, Jurkat wild type cells and transfected with scramble sequences were used.

### Cell cultures, proliferation and aggregation

Human epithelial (4/5) [14] and embryonic (HEK293) kidney cells were cultured in DMEM 50% F12 medium supplemented with 10% FBS. Jurkat cells and EBV transformed B lymphocytes (LCLs) [6] were cultured in RPMI 1640 medium with 10% FBS, while the medium for Human T lymphocytes (TLs) was supplemented with 50 U/mL IL-2. All cell types were maintained at 37 °C by a humidified (95% air, 5% CO_2_) incubator.

Cell proliferation was analysed by direct counting using a Burker chamber. T lymphocytes were seeded in 200 μL (2.5 × 10^4^ cell/mL) in 96 multiwell plates and cultured for 24 and 48 h in complete medium. Cell number was calculated performing three different cell counts in at least three separate cell cultures for every sample.

For the analysis of cell aggregation, cells were seeded in 2 mL at a density of 2.5 × 10^5^ cell/mL in 24 multiwell plates. Then, cells were cultured for 1 day in complete medium supplemented with 50 U/mL IL-2. TL clump size was evaluated using an inverted phase-contrast microscope (Nikon Eclipse TE200, Melville, NY, USA). Images were acquired with a CCD camera COOLSNAP (RS Photometrics, Tucson, Arizona, USA) and processed by ImageJ software.

### Chemotaxis analysis

Basal chemotaxis was evaluated with a 48-well microchemotaxis chamber (BioProbe, Milan, Italy) in polymorphonuclear neutrophils by adding 1 mg/mL BSA (Sigma-Aldrich) in the lower compartment of the chamber. BSA was diluted in KRPG-A buffer. Assays were performed in duplicate under each experimental condition. Data were expressed in terms of chemotactic index (CI), calculated through the following ratio: (migration toward test attractant minus migration toward the buffer)/migration toward the buffer [15].

### Western blotting

Cells were washed twice with ice-cold D-PBS containing protease inhibitors (Sigma-Aldrich) and collected by centrifugation at 800 *g* for 10 min. The pellet was resuspended in a single detergent lysis buffer (10 mM TRIS-HCl, pH 7.5, 10 mM NaCl, 3 mM MgCl_2_, 1% v/v triton X-100 supplemented with complete protease and phosphatase inhibitors) for 30 min on ice. After centrifugation at 10000 *g* for 10 min, total cell lysates (50 μg) were electrophoresed in 4–10% gradient SDS-polyacrylamide gel and transferred onto nitrocellulose filters (EuroClone) for 2 h in transfer buffer (25 mM TRIS, 192 mM glycine, 20% methanol, pH 8.3). Filters were blocked overnight at 4 °C in 5% non-fat dry milk in D-PBS with 0.05% Tween 20® and then processed for immunoblotting with the appropriate primary antibody. After 2 h of incubation, three washes with D-PBS with 0.05% Tween20® were performed and then the nitrocellulose membranes were incubated with the respective HRP-conjugated secondary antibody. After three washes with D-PBS with 0.05% Tween 20®, immunobands were visualised by autoradiography with enhanced chemiluminescence system (EuroClone). Band intensity was quantitatively detected by film scanning with the GS-700 Imaging Densitometer (BIO-RAD, Italy). Relative protein abundance was calculated as the ratio between the protein of interest and β-actin [16].

### Calcium measurements

Fluorescence measurements were performed either in saline solution containing 125 mM NaCl, 5 mM KCl, 1 mM MgSO_4_, 1 mM Na_2_HPO_4_, 5.5 mM glucose, 5 mM NaHCO_3_, 1 mM CaCl_2_ and 20 mM HEPES (pH 7.4 with NaOH) or in a Na^+^-free saline solution containing 300 mM sucrose, 1 mM MgSO_4_, 1 mM K_2_HPO_4_, 5.5 mM glucose, 1 mM CaCl_2_ and 20 mM HEPES (pH 7.4 with KOH). Changes of intracellular Ca^2+^ concentration in T lymphocytes or Jurkat cells were measured with Fura 2-AM using an LS50 Perkin Elmer fluorometer (Perkin Elmer Ltd., Beaconsfield, UK). First, cells (3 × 10^6^/mL) were resuspended in 0.9% NaCl solution with 1 μM Fura 2-AM and 250 μM sulfinpyrazone (Sigma-Aldrich) and incubated at 37 °C for 30 min. Cells were then washed and resuspended in the Na^+^-free saline solution. Intracellular calcium changes were determined in a thermostatic, magnetically stirred cuvette with 340/380 excitation ratio at an emission wavelength of 505 nm. Cells were stimulated with 2 μM PAF or with 100 μM ATP, successively treated with 2 μM ionomycin and finally diluted with 250 mM EGTA solution (Sigma-Aldrich) to chelate all calcium released [17]. For the measurements of intracellular calcium levels from the stores, Jurkat cells (3 × 10^6^/mL) were loaded with Fura 2-AM and maintained in saline free calcium solution containing 100 μM EGTA. Next, cells were stimulated with 100 mM ATP and, when the fluorescence returns to basal level, a 4 mM calcium solution was added to saline buffer in order to detect calcium influx through the plasma membrane. Changes in calcium concentration were calculated as described above.

### Statistical analysis

The statistical analysis was performed using Student’s t or ANOVA test, as appropriate. Data were expressed as mean ± standard deviation and differences were considered significant at *p* < 0.05.

## Results

### TRPP2 expression in control and ADPKD T lymphocytes

**Fig. 1:**
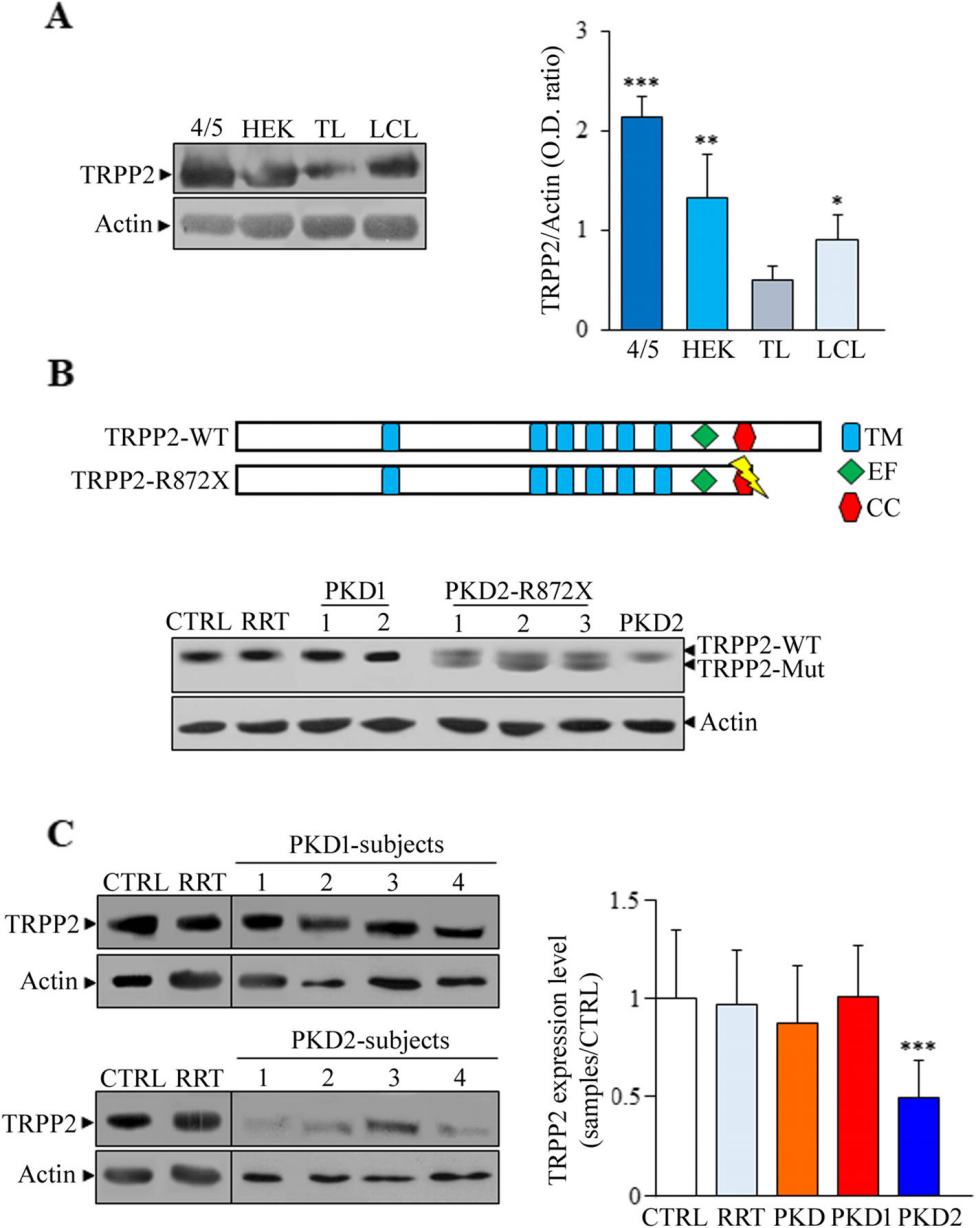
Analysis of TRPP2 in normal and ADPKD cells by Western blotting. **a** TRPP2 is expressed in two different normal kidney cell lines (4/5 and HEK293) as well as in T and B lymphocytes generated by healthy controls (TL and LCL, respectively). TRPP2 expression, is lower in T lymphocytes compared with the other cell types (0.49 ± 0.12 for TL, 0.89 ± 0.21 for LCL, 1.31 ± 0.36 for HEK and 2.14 ± 0.16 for 4/5 cells. TL vs LCL, HEK and 4/5: * *p* < 0.05, ** *p* < 0.01 and *** *p* < 0.001, respectively). Data represent the mean ± standard deviation obtained from two different experiments in duplicate. Statistical significance was calculated by using the unpaired t-test. **b** T lymphocytes derived from ADPKD2 subjects carrying R872X mutation synthesise a stable truncated protein detectable by Western blotting. TM = transmembrane domain; EF = EF hand domain; CC = coiled coil motif. **c** TRPP2 expression is lower in T lymphocytes of ADPKD2 subjects compared with non-genetically defined ADPKD, ADPKD1 and control subjects (0.50 ± 0.18 in PKD2, 1.01 ± 0.26 in PKD1, 0.88 ± 0.29 in PKD, 0.97 ± 0.28 in RRT and 1.0 ± 0.35 in CTRL cells. PKD2 vs CTRL: ***p < 0.001). CTRL = healthy controls (*n* = 10); RRT = non-ADPKD subjects undergoing renal replacement therapy (*n* = 14); PKD = non-genetically defined ADPKD subjects (*n* = 12); PKD1 = ADPKD1 subjects (*n* = 11); PKD2 = ADPKD2 subjects (*n* = 16).TRPP2 values were calculated as ratio between the band intensity of TRPP2 and β-actin. Bars of graph C represent the values of TRPP2 (mean ± standard deviation) calculated as ratio between TRPP2 levels of different samples and the average of those obtained from healthy controls (CTRL). The values of TRPP2 and PC1 expression in analysed ADPKD subjects and controls are inserted in Additional file 2: Table S2

Because the expression and function of TRPP2 in human ADPKD T lymphocytes is not well defined, we have investigated TRPP2 in these cells. We have isolated T lymphocytes from *PKD1*, *PKD2* and not genetically determined ADPKD patients as well as from non-ADPKD subjects undergoing renal replacement therapy (RRT) and healthy controls. First of all, T lymphocytes were characterized for CD3 expression and activation. These cells express the CD3 receptor (Additional file 3: Figure S1A) and their activation by using different doses of anti-CD3 antibody leads to the release of intracellular calcium (Additional file 3: Figure S1B). The analysis of TRPP2 expression (Fig. 1a) showed that this protein is expressed in T lymphocytes, but at lower levels compared with 4/5 and HEK293 kidney cells as well as EBV-transformed B lymphocytes (LCLs). The expression analysis of ADPKD patients carrying R872X mutation of *PKD2* showed the presence of both wild type and mutated TRPP2 proteins. In fact, the lower molecular weight band is consistent with the mutated protein (Fig. 1b). Nevertheless, the expression levels of TRPP2 in TLs of ADPKD2 patients were significantly lower than in TLs of control subjects as well as in those of ADPKD patients not genetically defined and ADPKD1 (Fig. 1c), suggesting that *PKD2* mutation could affect the stability of TRPP2. Interestingly, we have analysed the expression of TRPP2 in a subject carrying two variants in *PKD1* and one in *PKD2* (Table 1, patient number 90). The variants of *PKD1* are R2765C and R3348Q substitutions, but only the latter could be pathogenic, while the *PKD2* lesion is the frameshift mutation A365fs, which is considered pathogenic (PKD Mutation Database, http://pkdb.mayo.edu). Consistently, the expression of TRPP2 in TLs of this subject was lower compared with control cells (PKD2 sample number 3 of Fig. 1c), indicating that this mutation affects the expression of TRPP2. On the contrary, the expression of PC1 was unchanged (data not shown).

**Table 1:** *PKD2* variants in 21 ADPKD2 patients

Patient number	Family ID	Mutation (cDNA)	Protein change	TRPP2 expression (ADPKD2/controls)	Dialysis (age)	Hypertension	Extra-renal manifestations	Pathogenicity of *PKD2* mutation
90	PKD2MO65	8293C > T and 10043G > A in *PKD1*; IVS4 + 1G > A in *PKD2*	In PC1: Arg2765Cys and Arg3348Gln. In TRPP2: Ala365fs	0.74	no	no	none	definitely pathogenic
91	PKD2MO50	2614C > T	Arg872X	0.55	no	yes	Pancreatic and liver cysts	definitely pathogenic
92	PKD2MO50	2614C > T	Arg872X	0.78	no	yes	mitral prolapse	definitely pathogenic
93	PKD2MO50	2614C > T	Arg872X	0.31	yes (72)	yes	none	definitely pathogenic
94	PKD2MO50	2614C > T	Arg872X	0.7	no	no	none	definitely pathogenic
95	PKD2MO87	ivs3 + 1G > T	Phe282fs	0.86	yes (59)	yes	polycystic liver, diverticulosis of colon	definitely pathogenic
96	PKD2MO87	ivs3 + 1G > T	Phe282fs	0.43	yes (50)	yes	polycystic liver	definitely pathogenic
97	PKD2MO87	ivs3 + 1G > T	Phe282fs	0.51	no	yes	none	definitely pathogenic
98	PKD2MO87	ivs3 + 1G > T	Phe282fs	NA	yes (62)	yes	none	definitely pathogenic
99	PKD2MO87	ivs3 + 1G > T	Phe282fs	NA	yes (60)	yes	polycystic liver	definitely pathogenic
100	PKD2MO21	ivs5 + 5G > A, ivs9 2020-2089_86del	unknown	NA	no	yes	polycystic liver	unknown
101	PKD2MO21	ivs5 + 5G > A, ivs9 2020-2089_86del	unknown	NA	no	yes	none	unknown
102	PKD2MO21	ivs5 + 5G > A, ivs9 2020-2089_86del	unknown	NA	no	yes	none	unknown
103	PKD2MO29	1158 T > A	Tyr386X	0.39	no	no	polycystic liver	definitely pathogenic
104	PKD2FE15	1158 T > A	Tyr386X	0.47	yes (67)	yes	none	definitely pathogenic
105	PKD2FE15	1158 T > A	Tyr386X	0.31	no	yes	none	definitely pathogenic
106	PKD2FE15	1158 T > A	Tyr386X	0.55	yes (55)	no	cerebral aneurism	definitely pathogenic
107	PKD2FE15	1158 T > A	Tyr386X	0.39	yes (69)	no	none	definitely pathogenic
108	PKD2FE5	858delC	Ser286fs30X	0.27	yes (82)	no	myocardiosclerosis	definitely pathogenic
109	PKD2FE5	858delC	Ser286fs30X	0.35	yes (73)	no	obstructive vascular disease	definitely pathogenic
110	PKD2FE5	858delC	Ser286fs30X	0.39	yes (66)	no	polycystic liver, myocardiosclerosis and cerebral ictus	definitely pathogenic

ADPKD2 patient numbering is referred to Additional file 1: Table S1

The age of ADPKD2 patients is ranging from 26 to 89 years. TRPP2 expression and clinical parameters are indicated. Values of TRPP2 were calculated as ratio between the levels of TRPP2 in ADPKD2 patients and healthy controls

### PKD2 mutations affect renal outcome in ADPKD2 patients

It is known that mutations of PKD1 gene affect the clinical outcome of ADPKD1 patients [18], therefore, we have analysed the possible correlation among the type of *PKD2* mutation and phenotypic outcome in our ADPKD2 cohort. As shown in Table 1, this study reveals a strong variability linked to the type of mutation as well as high intra familial differences. Both *PKD2* mutations in PKD2MO50 and PKD2MO21 families are not associated with severe renal complications, but likely with the high blood pressure. Conversely, the mutation F282 fs causes severe disease with early ESRD and hypertension similarly to ADPKD1 patients. The mutation Y386X shows an elevated inter and intra familial phenotypic variability, in particular, for the extra-renal manifestations. Finally, the mutation S286fs30X is linked to a mild form of disease with late ESRD, but causes severe extra-renal manifestations including cardiovascular dysfunctions.

### Analysis of intracellular calcium in TRPP2-deficient blood cells

**Fig. 2:**
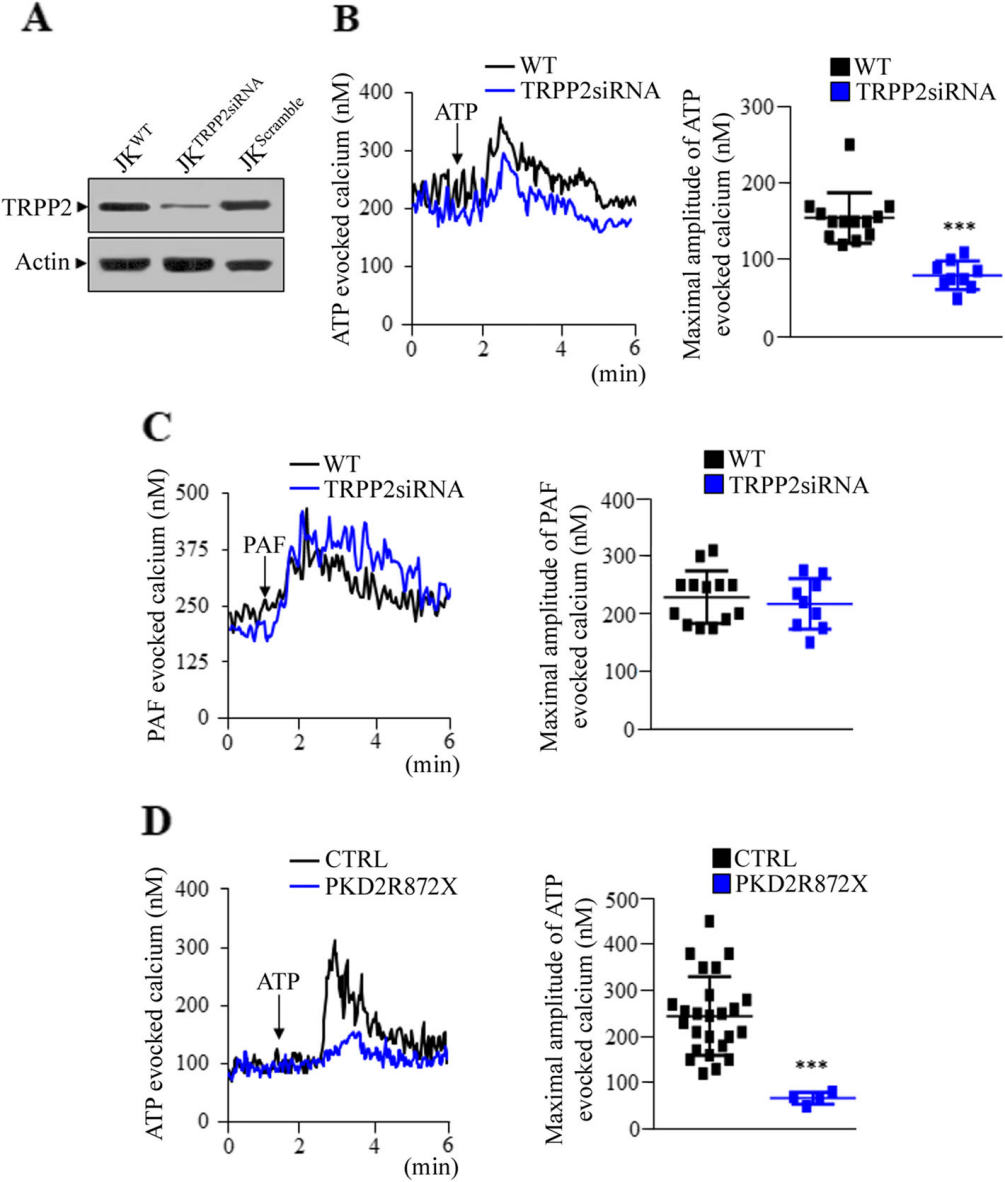
Analysis of ATP- and PAF-evoked calcium in TRPP2 silenced Jurkat cells and ADPKD2 subjects currying the R872X mutation. **a** The expression of TRPP2 was evaluated by Western blotting in wild type Jurkat cells and transfected with TRPP2-siRNA plasmid or with scramble sequences. The calcium levels were measured after ATP (**b**) or PAF (**c**) stimulation in control (WT) and in *PKD2* silenced (TRPP2-siRNA) Jurkat cells by Fura 2-AM method. The application of 100 μM ATP causes a significant reduction of cytosolic calcium release in TRPP2 silenced cells compared with control cells (80.1 ± 18.4 for TRPP2-siRNA and 154.9 ± 32.7 for WT: ***p < 0.001). No significant differences in calcium release between control and TRPP2 silenced Jurkat cells after 2 μM PAF stimulation were observed. For both ATP- and PAF-evoked calcium were performed 13 and 9 measurements in WT and TRPP2siRNA Jurkat cells, respectively. **d** The intracellular calcium release after ATP stimulation is lower in T lymphocytes of ADPKD2 subjects currying the R872X mutation than in CTRL (67.5 ± 12.58 for *PKD2*-R872X and 244.8 ± 85.5 for CTRL: ***p < 0.001). The maximal calcium concentration after ATP or PAF stimulation was calculated as Δ (delta) obtained from the maximal value minus the basal one. Data are expressed as mean ± standard deviation calculated from at least two different experiments in duplicate, while for *PKD2-*R872X T lymphocytes values represent the mean ± standard deviation of two experiments. *PKD2-*R872X = ADPKD2 subjects currying R872X mutation (*n* = 4). CTRL = healthy controls (*n* = 25). Data of ATP- and PAF-evoked calcium detected in WT and *PKD2* silenced Jurkat cells are inserted in Additional file 2: Table S2

**Fig. 3:**
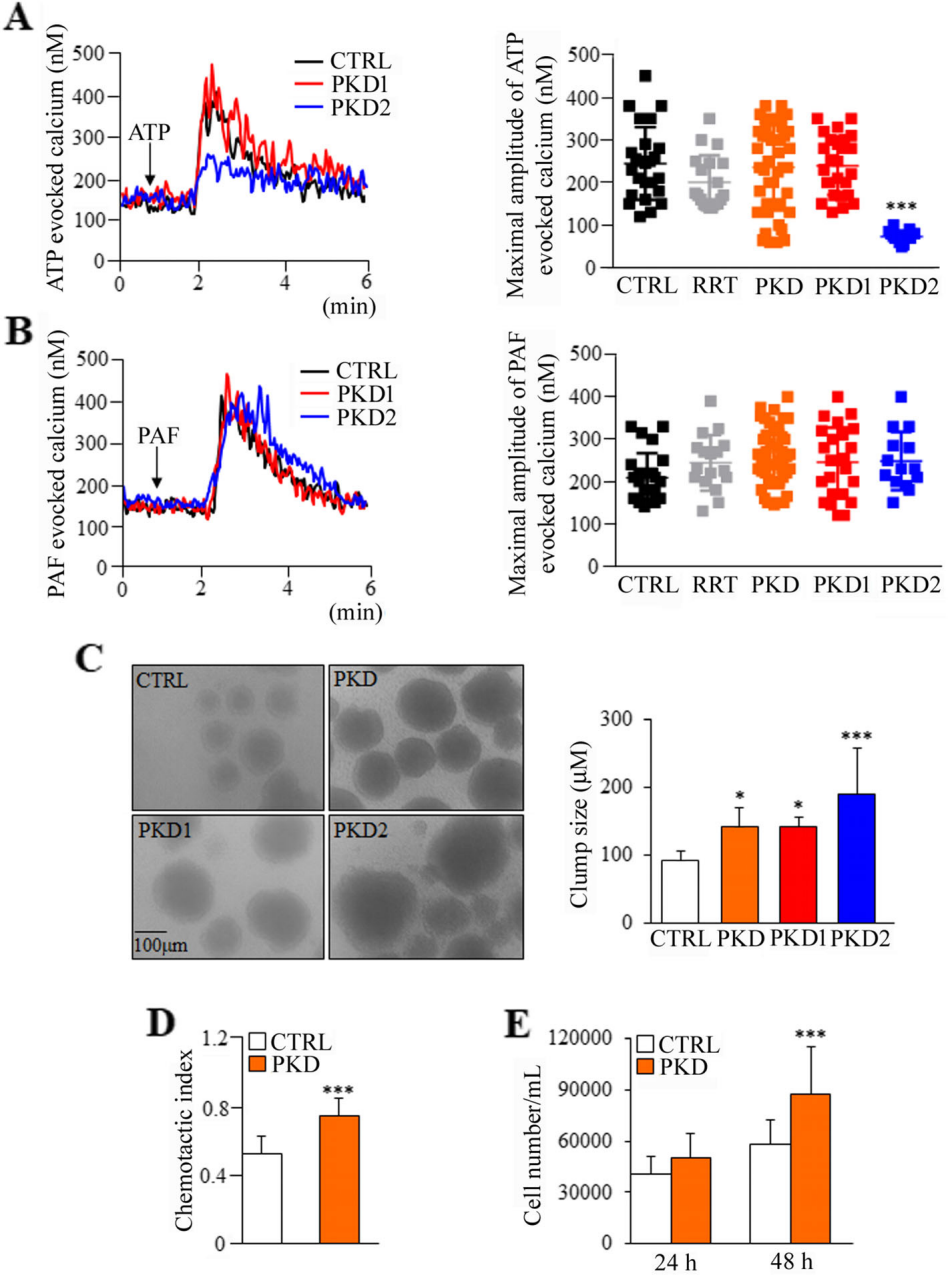
Analysis of intracellular calcium release, cell aggregation, chemotactic index and cell proliferation in normal and ADPKD T lymphocytes. **a** ATP-evoked calcium is lower in T lymphocytes of ADPKD2 patients compared with non-genetically defined ADPKD, ADPKD1 and control subjects (73.67 ± 13.02 for PKD2 and 244.8 ± 85.51 for CTRL: ***p < 0.001). CTRL (*n* = 25), RRT (*n* = 19), PKD (*n* = 44), PKD1 (*n* = 26) and PKD2 (*n* = 15). **b** No changes in PAF-evoked calcium release were observed among controls and ADPKD T lymphocytes. CTRL (*n* = 24), RRT (*n* = 17), PKD (*n* = 49), PKD1 (*n* = 24) and PKD2 (*n* = 14). The maximal calcium concentration in response to ATP or PAF in T lymphocytes was calculated as described in Fig. 2. **c** ADPKD T lymphocytes form clamps greater in size as compared with those of control (190 ± 68 for PKD2, 143 ± 14 for PKD1, 141 ± 28 for PKD and 91 ± 15 for control cells. PKD and PKD1 vs. CTRL: *p < 0.05, PKD2 vs. CTRL: ***p < 0.001). Images were acquired by using an inverted phase-contrast microscope equipped with a CCD camera. **d** In basal conditions, ADPKD T lymphocytes show a greater chemotactic index compared with control cells (0.75 ± 0.1 for PKD and 0.53 ± 0.1 for CTRL: ***p < 0.001). **e** After 48 h of culture, ADPKD T lymphocytes grew faster than control cells (87,725 ± 27,173 for PKD and 58,003 ± 14,467 for CTRL: ***p < 0.001). CTRL = healthy controls; RRT = non-ADPKD subjects undergoing renal replacement therapy; PKD = non-genetically determined subjects; PKD1 = *PKD1*-related subjects; PKD2 = *PKD2*-related subjects. Data are expressed as mean ± standard deviation calculated from at least two different experiments in duplicate. All values of calcium measurements after ATP and PAF stimulation in control and ADPKD T lymphocytes are shown in Additional file 2: Table S2

TRPP2 functions as a non-selective calcium permeable channel activated by mechanical bending in primary cilia that regulates calcium entry into kidney epithelial cells [19]. The channel activity of TRPP2 was also observed in other cell compartments, such as the plasma membrane and endoplasmic reticulum [5, 20]. In addition, we have previously reported that the silencing of TRPP2 in HEK293 kidney cells led to the reduction of TRPP2-dependent channel activity [12]. However, little is known on the function of TRPP2 in T lymphocytes, therefore, we have silenced *PKD2* in immortalized T lymphocytes (Jurkat cells), in order to evaluate the channel activity of TRPP2. As expected, the silencing of *PKD2* in Jurkat cells decreased the expression of TRPP2 protein compared to those transfected with scramble sequences or wild type cells (Fig. 2a). Moreover, the downregulation of TRPP2 caused a significant reduction of ATP-evoked intracellular calcium concentration compared with wild type cells (Fig. 2b). No significant change in intracellular calcium content after PAF stimulation in TRPP2 silenced Jurkat cells as compared to wild type cells was observed (Fig. 2c). Interestingly, the mutation R872X, which is classified as a pathogenic lesion, produces a stable truncated TRPP2 protein in all subjects carrying this mutation (Fig. 1b). In particular, this mutation truncates the coiled coil domain (aa 832–895) located in the C-terminal tail of TRPP2 that is crucial for the protein assembly and for its interaction with other calcium channels [21]. Consistently, R872X ADPKD2 T lymphocytes show reduced levels of intracellular calcium after ATP stimulation compared with those produced from CTRL subjects (Fig. 2d). Importantly, in T lymphocytes derived from other ADPKD2 subjects that express reduced levels of TRPP2, a lower intracellular calcium release than in CTRL cells after ATP stimulation was observed (Fig. 3a). On the contrary, the treatment with ATP in T lymphocytes generated by patients linked to *PKD1* did not cause changes in intracellular calcium concentration (Fig. 3a). As observed in Jurkat cells (Fig. 2c), the stimulation with PAF of any kind of ADPKD T lymphocytes did not significantly modify calcium levels compared to RRT subjects and healthy controls (Fig. 3b). In order to evaluate how TRPP2 affects intracellular calcium levels in T lymphocytes, ATP-evoked calcium was analysed, firstly, in calcium free conditions and, subsequently, reintroducing calcium ions to the saline buffer. In absence of calcium, the stimulation with ATP caused a lower depletion of calcium from the stores in *PKD2* silenced Jurkat cells compared with wild type (Additional file 3: Figure S1C). After calcium reintroduction, no significant changes in calcium elevation among Jurkat cells downregulated for *PKD2* and wild type cells were observed (Additional file 3: Figure S1C).

### Polycystin dysfunction increases homotypic aggregation of T lymphocytes, enhances neutrophil chemotaxis and stimulates cell growth in T lymphocytes

Cell aggregation can play important roles in different biological processes, including the immune response. In fact, homotypic cell aggregation is involved in the regulation of several immune functions, such as cellular localisation and T lymphocyte activation [22]. We have observed an increased size of homotypic cell aggregates in T lymphocytes of ADPKD patients compared with control cells (Fig. 3c). In particular, this phenomenon is greater in T lymphocytes of ADPKD2 subjects (Fig. 3c) suggesting that TRPP2 loss of function may have an important role in cell-cell interaction of T lymphocytes. Leucocyte aggregation may be activated by different pathways including the signal modulated by mitogen-activated protein kinases (MAPKs), that is also involved in the abnormal cell proliferation in ADPKD kidney cystic cells [1, 23]. These molecules could recruit leucocytes, including T lymphocytes, increasing interstitial inflammation and promoting cyst growth and expansion. Actually, increased chemotaxis of neutrophils derived from ADPKD subjects compared with those generated by healthy controls in basal conditions of culture (cells cultured in presence of 1 mg/mL of BSA) has been observed (Fig. 3d). Moreover, T lymphocytes produced by ADPKD patients, after 48 h of culture, exhibit higher cell proliferation rate compared with those derived from healthy controls (Fig. 3e).

### ADPKD T lymphocytes show increased activation of ERK and mTOR kinases, as well as enhanced expression of NFkB and MIF

**Fig. 4:**
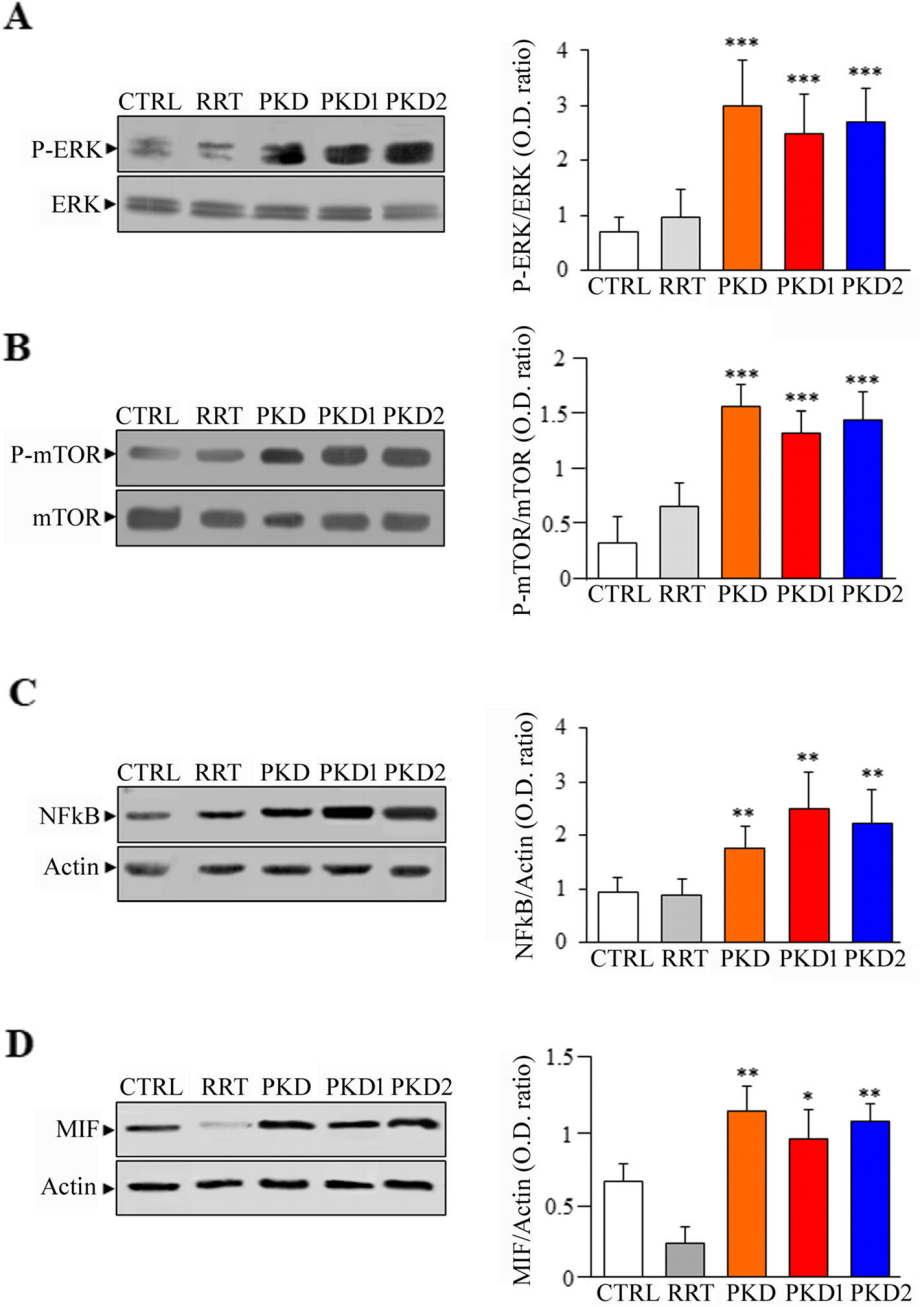
Analysis of p-ERK, p-mTOR, NFkB and MIF expression in ADPKD T lymphocytes. **a** ERK phosphorylation is increased in ADPKD compared with control cells (2.7 ± 0.6 for PKD2; 2.5 ± 0.7 for PKD1; 3 ± 0.8 for PKD; 0.95 ± 0.51 for RRT; 0.7 ± 0.26 for CTRL. PKD2, PKD1 and PKD vs. CTRL: ***p < 0.001). **b** The activation of mTOR is greater in ADPKD than in CTRL T lymphocytes (1.43. ± 0.25 for PKD2; 1.34 ± 0.2 for PKD1; 1.55 ± 0.21 for PKD; 0.65 ± 0.21 for RRT; 0.31 ± 0.24 for CTRL. PKD2, PKD1 and PKD vs. CTRL: ***p < 0.001). **c** ADPKD T lymphocytes show increased levels of NFkB protein compared with control cells (2.23. ± 0.61 for PKD2; 2.49 ± 0.68 for PKD1; 1.74 ± 0.40 for PKD; 0.89 ± 0.30 for RTT; 0.94 ± 0.27 for CTRL. PKD2, PKD1 and PKD vs. CTRL: **p < 0.01). **d** The expression of MIF is higher in ADPKD T lymphocytes than in controls (1.06 ± 0.12 for PKD2; 0.94 ± 0.20 for PKD1; 1.13 ± 0.17 for PKD; 0.23 ± 0.10 for RRT; 0.65 ± 0.13 for CTRL. PKD2 and PKD vs CTRL: **p < 0.01; PKD1 vs CTRL: *p < 0.05). CTRL = healthy controls; RRT = non-ADPKD subjects undergoing renal replacement therapy; PKD = non-genetically determined subjects; PKD1 = *PKD1*-related subjects; PKD2 = *PKD2*-related subjects. The phosphorylation levels were calculated as the ratio between band intensity of the phosphorylated form and total protein, while the expression of NFkB and MIF was calculated as ratio among the band relative to these proteins and beta-Actin. Data are expressed as mean ± standard deviation calculated from at least two different experiments in duplicate

Besides MAPKs, other signalling pathways are dysregulated in ADPKD, including PI-3 K, ERK and mTOR, which are associated with the enhanced cell proliferation of kidney epithelial cystic cells [24]. These signals deeply contribute to the development and progression of ADPKD, and the inhibition of these pathways reduces cyst progression particularly in animal models of ADPKD [1]. As reported in ADPKD epithelial cystic cells [25], ADPKD T lymphocytes derived from both ADPKD1 and ADPKD2 patients show increased levels of ERK kinases compared with cells isolated from non-ADPKD subjects and healthy controls (Fig. 4a). In addition, the signalling mediated by mTOR kinase, which is strongly involved in ADPKD pathology, is abnormally activated in T lymphocytes of ADPKD patients compared with controls (Fig. 4b). Moreover, the activation of NFkB that stimulates the expression of proinflammatory cytokines in subjects with chronic kidney disease (CKD) [26] resulted greater in ADPKD T lymphocytes compared with control cells (Fig. 4c). Consistently, also the macrophage migration inhibitory factor (MIF) that promotes the expression of inflammatory cytokines [24] was found significantly increased in T lymphocytes derived from ADPKD patients as compared to those generated by healthy and RRT controls (Fig. 4d).

## Discussion

The role of TRPP2 in human ADPKD T lymphocytes was not well investigated. In particular, the expression of polycystins in T lymphocytes may affect the processes mediated by these cells, such as immune response and inflammation that are altered in ADPKD subjects. Therefore, the mutation of both PC1 and TRPP2 could contribute to the activation of these processes promoting disease progression [27]. We have observed that the mutation Arg872X of TRPP2, localized at the end of *PKD2,* generates a TRPP2 truncated protein that could interfere with the wild type causing the dysfunction of the polycystin complex. In fact, the mutation Arg872X leads to the reduction of ATP-evoked calcium in T lymphocytes isolated from patients carrying this mutation. Consistently, different studies using TRPP2 mutated/truncated proteins show that TRPP2 defective proteins may be stable and could translocate to the cilia in epithelial cells leading to the impairment of channel activity [28]. Moreover, the reduced expression of TRPP2 (< 50%), observed in the most of ADPKD2 T lymphocytes, suggests that the mutated form of this protein could affect the correct channel assembly leading to its degradation by lysosomal and proteasome systems, as observed in other cell types [29, 30]. The low expression of TRPP2 may indicate the presence of *PKD2* mutation, therefore the analysis of TRPP2 expression in not genetically defined ADPKD patients could lead to the detection of ADPKD type 2 subjects. In this regard, we observed lower expression levels of TRPP2, but not of PC1, in T lymphocytes of a patient carrying a *PKD2* frameshift mutation (A365fs) and two *PKD1* variants (R2765C and R3348Q). This finding suggests that the *PKD2* mutation could affect TRPP2 stability, in fact, this frameshift mutation is considered highly pathogenic. Consistently, the clinical parameters of the patient carrying this mutation show a mild form of disease, indicating the possible linkage to *PKD2* lesion (Table 1). Multiple mutations of *PKD1* and *PKD2* in a single ADPKD subject are not surprising, because they have been already described [31]. In fact, an ADPKD patient showed a sporadic splice variant of *PKD1* and a homozygous missense mutation of *PKD2* that caused the perinuclear cytoplasmic distribution of the mutated protein and reduced channel activity [31]. As observed for PKD1 [18], the type of PKD2 mutation deeply affects renal and extra-renal outcome of ADPKD2 patients (Table 1), furthermore the high familial variability of clinical symptoms observed in these patients suggests the existence of modifiers factors that might affect disease progression. These observations confirm previous studies that show the possible correlation among the variable penetrance in ADPKD population with environmental and genetic modifiers [32].

The dysfunction of TRPP2 in T lymphocytes causes the reduction of intracellular calcium release after ATP stimulation. Thus, also in these cells, TRPP2 may function as calcium channel able to regulate the intracellular calcium entry. Consistently, it was found that the silencing of *PKD2* reduces the channel activity as well as abolishes the Ca^2+^-dependent currents in HEK293 and LLC-PK1 renal cells, respectively [12, 33]. Surprisingly, ATP stimulation of T lymphocytes derived from ADPKD1 patients does not change the intracellular calcium concentration compared with CTRL cells, likely because, in ADPKD1 T lymphocytes, the levels of PC1 protein are unchanged (data not shown). The stoichiometric ratio between PC1 and TRPP2 could be crucial for the regulation of channel activity in response to ATP in kidney cells as well as in T lymphocytes. In this regard, it was reported that the increased expression of TRPP2, interacting with IP3R, enhances the intracellular Ca^2+^ release in MDCK kidney cells. Conversely, the overexpression of PC1, likely removing TRPP2 from IP3R, reduces the activity of this receptor channel, and consequently, causes the decrease of ATP-evoked intracellular calcium. Taken together these observations suggest that TRPP2 is a calcium channel that may modulate calcium release from ER in kidney cells [34]. Consistently, we have detected lower intracellular calcium levels after ATP stimulation in calcium free conditions in *PKD2* silenced Jurkat cells compared with wild type, while no differences in calcium content after calcium reintroduction were found. Therefore, in T lymphocytes TRPP2 seems to work mainly in endoplasmic reticulum, likely, interacting with IP3R and contributing to the calcium release from the stores, as already described in kidney cells [35]. Previously, we have reported that PAF stimulation of B-LCLs derived from ADPKD2 patients showed lower intracellular calcium levels compared with ADPKD1 and control cells [6]. In T lymphocytes, there was no statistically significant reduction of calcium concentration after PAF application in ADPKD2 cells compared to ADPKD1 or control cells (Fig. 3b). The different PAF response between B and T lymphocytes may be due to the different features of TLs and B-LCLs that could affect their sensitivity to PAF stimulation. On the other hand, B-LCLs are Epstein-Barr transformed cells, while TLs are stimulated by IL-2 only. Alternately, we speculate that the calcium response after PAF stimulation can be greater in T lymphocytes than in B-LCLs, leading to the inhibition of TRPP2. In fact, it was reported that the release of high levels of intracellular calcium could inhibit this cation channel [36]. Moreover, T lymphocytes express lower levels of TRPP2 than B-LCLs (Fig. 1a), therefore, TRPP2 could be inhibited by PAF-evoked calcium in T lymphocytes, but not in B-LCLs. These observations could also explain the different calcium response detected in ADPKD2 T lymphocytes after ATP or PAF stimulation. As described above, the treatment with ATP induced lower intracellular calcium levels in ADPKD2 T lymphocytes compared with control cells, but no changes in PAF-evoked calcium were observed. Both ATP and PAF may interact with receptors that lead to IP3 production and calcium release from ER through the activation of IP3R channels. We presume that the ATP stimulation in T lymphocytes may cause the release of intracellular calcium levels that prevent the inhibition of TRPP2 channel.

Taken together, these observations suggest that TRPP2 could function as a channel complex that regulates ATP-evoked intracellular calcium release in T lymphocytes.

It is known that interstitial inflammation is one of the causes of cystic progression in ADPKD, and cell-cell adhesion may also function as a mechanism to recruit leucocytes and contribute to the development of the inflammatory process [23]. Consistently, we have found increased neutrophil chemotaxis, T lymphocyte aggregation and enhanced proliferation in cells derived from ADPKD patients. These processes could be involved in the development of inflammation and affect disease progression. Neutrophil chemotaxis may also be driven by the macrophage migration inhibitory factor (MIF), which is expressed in neutrophils and might promote neutrophil trafficking in inflammatory processes [37]. Moreover, in kidney diseases, MIF contributes significantly to macrophage and T lymphocyte accumulation contributing to progressive renal injury [38]. In addition, MIF was upregulated in cyst-lining epithelial kidney cells of *Pkd1*-deficient mice as well as in the cyst fluid of human ADPKD kidneys [24]. MIF regulates different cellular activities, including the modulation of cell proliferation, differentiation, cell cycle and metabolism through the activation of different signalling pathways [24, 38, 39]. Thus, the loss of function of *PKD* genes may enhance the chemotaxis, cell proliferation and aggregation increasing the inflammatory process commonly observed in ADPKD cysts. Actually, we have observed that T lymphocytes isolated from ADPKD patient express higher levels of MIF than T lymphocytes derived from healthy and RRT controls. These data confirm that MIF may contribute to inflammation in ADPKD.

MIF activation may promote cell proliferation by activating both ERK, mTOR, signaling in ADPKD cystic cells [24]. Consistently, we have observed the abnormal activation of ERK and mTOR kinases in ADPKD T lymphocytes. Thus, a common mechanism independent from cell type could be activated by *PKD1 and PKD2* mutation leading to the activation of these protein kinases. As observed in cells with loss of function of PC1 [25], also TRPP2 dysfunction causes the abnormal activation of ERK pathway that is directly involved in increased cell growth, a typical hallmark of ADPKD [40]. Moreover, other studies describe that mTOR signalling is most activated in Pkd2WS25/2 mice, a model for ADPKD type 2, than in wild type mice [41], confirming the involvement of mTOR in abnormal cell proliferation of ADPKD2 cells. Interestingly, in T lymphocytes of ADPKD patients, we have also observed an increased expression of NFkB, as already reported for ADPKD kidney cells [42]. Therefore, the enhanced expression of NFkB found in ADPKD T lymphocytes could be involved in the development of inflammation. Consistently, the increase of NFkB protein expression consistent with inflammation in vascular endothelial cells of ADPKD subjects it has been detected [43].

Taken together, our findings confirm that PC1 and TRPP2 may control cell proliferation and migration by modulating the ERK, mTOR, NFkB and MIF signalling pathways in T lymphocytes and could contribute to inflammation, as occurs in kidney and endothelial cells.

## Conclusions

Here, we report that TRPP2 is markedly downregulated in ADPKD2 T lymphocytes compared with controls. Moreover, the mutation of *PKD2* that decreases TRPP2 expression causes the reduction of intracellular calcium entry after ATP stimulation in ADPKD2 T lymphocytes. Therefore, the combination between low levels of TRPP2 protein and the reduced ATP-dependent channel activity could represent a new tool able to detect not genetically identified ADPKD2 subjects or sporadic cases. As reported in kidney cells, the mutation of either *PKD1* or *PKD2* genes in T lymphocytes stimulates the activity of different signalling pathways including ERK, mTOR, NFkB and MIF leading to the activation of cell proliferation and aggregation. These proteins may also activate the inflammatory complex and contribute to kidney cyst enlargement and disease progression. Therefore, the targeting of molecules involved in inflammation could represent a new option to slow disease progression and preserve renal function in ADPKD patients.

## Additional files

Additional file 1: **Table S1.** “ADPKD patient cohort enrolled from four Nephrology Units of Emilia Romagna Region (Italy)”. This table contains information about ADPKD patients including clinical features. (XLSX 18 kb)

Additional file 2: **Table S2.** “Analysis of ATP- and PAF-evoked calcium as well as PC1 and TRPP2 expression in ADPKD patients”. In this table are inserted the values of PC1 and TRPP2 expression, data of ATP- and PAF-evoked calcium measured in ADPKD and control subjects as well as in wild type and *PKD2* silenced Jurkat cells. The type of *PKD* mutation (when known) is also shown. (XLSX 27 kb)

Additional file 3: **Figure S1.** “Characterization of T lymphocytes by CD3 expression and analysis of ATP-evoked calcium in WT and *PKD2* silenced Jurkat cells in Ca^2+^ free conditions”. In this figure, T lymphocytes have been characterized by CD3 expression and activation through the treatment with an anti-CD3 antibody. Moreover, the calcium release in response to ATP stimulation in calcium free conditions and after external calcium reintroduction is shown. (TIF 9041 kb)

## Data Availability

All data generated or analyzed during this study are included in this published article and its additional information files.

## Abbreviations

ADPKD: Autosomal dominant polycystic kidney disease
ATP: Adenosine triphosphate
CD3: Cluster of differentiation 3
EGF: Epidermal growth factor
EGTA: Ethylene glycol-bis(β-aminoethyl ether)-*N*,*N*,*N*′,*N*′-tetraacetic acid
ERK: Extracellular signal-regulated kinase
ESRD: End-stage renal disease
HEK293: Human embryonic kidney 293 cells
IL-2: Interleukin-2
KRPG: Krebs ringer phosphate glucose buffer
LCLs: Lymphoblastoid B cells
LLC-PK1: Epithelial-like pig kidney cell strain
MAPK: Mitogen-activated protein kinase
MEK: MAPK/ERK kinase
MIF: Macrophage-migration inhibitory factor
mTOR: Mammalian target of rapamycin
NFkB: Nuclear factor kappa-light-chain-enhancer of activated B cells
PAF: Platelet-activating factor
PBMC: Peripheral blood mononuclear cells
PBS: Phosphate-buffered saline
PC1: Polycystin-1
PHA: Phytohaemagglutinin
PI-3 K: Phosphatidylinositol 3-kinase
PMN: Polymorphonuclear neutrophils
RAF: Rapidly accelerated fibrosarcoma
RBC: Red blood cells
RPMI: Roswell park memorial institute medium
RTT: Renal replacement therapy
TLs: T lymphocytes
TRPP2: Transient receptor potential polycystic 2

## Footnotes

1 Renal replacement therapy (haemodialysis, peritoneal dialysis or renal transplant).
