# Supplementary figures and images for: TRPP2 dysfunction decreases ATP-evoked calcium, induces cell aggregation and stimulates proliferation in T lymphocytes
Source: BMC Nephrol. 2019 Sep 13;20:355. doi: 10.1186/s12882-019-1540-6 (PMC6743124; doi:10.1186/s12882-019-1540-6)

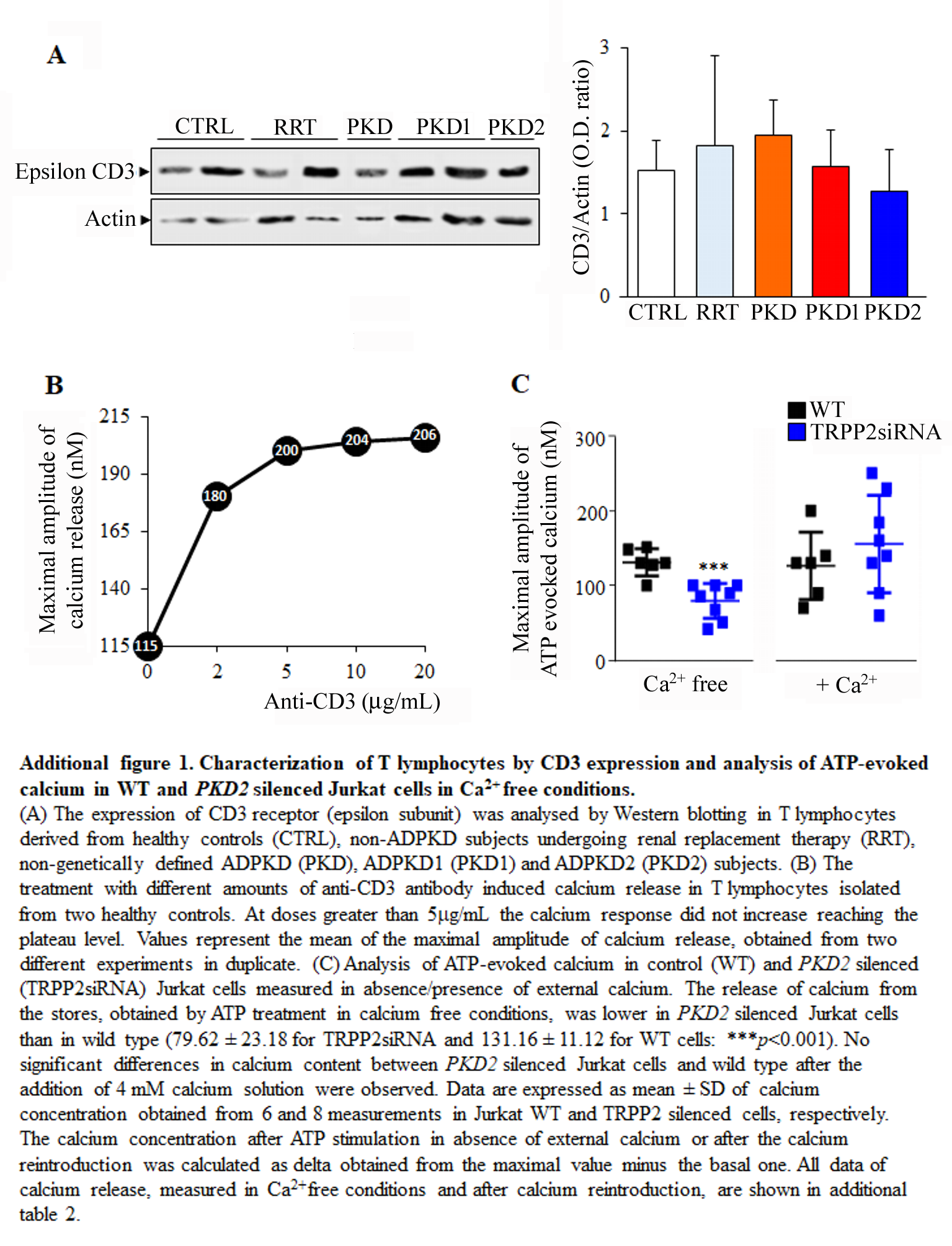

Supplement: Supplementary file 3 — Figure S1. “Characterization of T lymphocytes by CD3 expression and analysis of ATP-evoked calcium in WT and PKD2 silenced Jurkat cells in Ca2+ free conditions”. In this figure, T lymphocytes have been characterized by CD3 expression and activation through the treatment with an anti-CD3 antibody. Moreover, the calcium release in response to ATP stimulation in calcium free conditions and after external calcium reintroduction is shown. (TIF 9041 kb) [file 12882_2019_1540_MOESM3_ESM.tif]
